# First Insights Into Bacterial Gastrointestinal Tract Communities of the Eurasian Beaver (*Castor fiber*)

**DOI:** 10.3389/fmicb.2019.01646

**Published:** 2019-07-25

**Authors:** Rahadian Pratama, Dominik Schneider, Tim Böer, Rolf Daniel

**Affiliations:** ^1^Göttingen Genomics Laboratory, Department of Genomic and Applied Microbiology, Institute of Microbiology and Genetics, University of Göttingen, Göttingen, Germany; ^2^Department of Biochemistry, Faculty of Mathematics and Natural Sciences, Bogor Agricultural University (IPB University), Bogor, Indonesia

**Keywords:** Eurasian beaver, gut bacterial communities, cellulose, lignocellulose breakdown, Tax4Fun, herbivorous gut system

## Abstract

The Eurasian or European beaver (*Castor fiber*) is the second-largest living rodent after the capybara. It is a semi-aquatic animal known for building dams and lodges. They strictly feed on lignocellulose-rich plants and correspondingly harbor cellulolytic microbial communities in their digestive tract. In this study, the bacterial community composition, diversity, and functional profile of different gut compartments ranging from stomach to colon have been explored. A total of 277 bacterial operational taxonomic units (OTUs) at species level were obtained from the gut systems of two males (juvenile and subadult) and one subadult female beaver. In general, cecum and colon are dominated by Firmicutes and Actinobacteria. High abundance of Bacteroidetes was observed only in male juvenile beaver cecum and colon, suggesting that the bacterial composition changes with age. Within the cecum and colon, members of known cellulase-producing bacterial taxa including the families Ruminococcaceae, Lachnospiraceae, and Clostridiaceae 1 were detected. The presence of putative genes encoding cellulolytic and carbohydrate-degrading enzymes indicated also the degradation of recalcitrant plant material in both gut compartments. The bacterial community in the gut systems of the Eurasian beaver differed from that of the North American beaver. Higher abundance of Actinobacteria and lower abundances of Bacteroidetes were recorded in the Eurasian beaver. Similar differences were obtained to bacterial communities of termites and herbivorous animals such as bovine. The data presented in this study provides the first insight into bacterial communities in the gut system of the Eurasian beaver.

## Introduction

The main component of plant biomass, lignocellulose, consists mainly of the polysaccharides cellulose, hemicellulose, pectin, and lignin. Cellulose is a linear polymer of β-D-glucopyranose connected by β-1,4-linkages (O’Sullivan, 1997). The presence of these β-linkages results in inaccessibility for most animals as these lack enzymes capable to breakdown the β-linkages of cellulose. The degradation of some plant cell walls is difficult as in addition to cellulose they contain hemicellulose and lignin as structural components. Hemicellulose contains many types of polysaccharides, including xyloglucans, xylans, mannans and glucomannans, and β-(1→3,1→4)-glucans (Scheller and Ulvskov, 2010). In contrast to cellulose, hemicellulose is branched along its polymer structure. The microfiber of cellulose and hemicellulose is densely packed in lignin layers, which inhibit the activity of hydrolytic enzymes and support the structural stability of plant cell walls (Malherbe and Cloete, 2002). Lignocellulose composition varies depending on the plant type, with woody plants exhibiting a higher lignin content than herbaceous plants (Harris and Stone, 2008). Thus, it is more difficult to hydrolyze lignocellulose in woody plants than in herbaceous plants. Enzymes capable of degrading such recalcitrant material are of interest for industrial purposes, i.e., for efficient conversion of plant biomass into biofuel (Hood, 2016).

Several bacterial and fungal taxa are able to degrade lignocellulosic material. The fungus *Trichoderma reesei* strain RUT-C30 was extensively investigated for its cellulolytic capability (Peterson and Nevalainen, 2012). Other known cellulose-degrading fungi include *Aspergillus niger*, *Cladosporium cladosporioides*, *C. sphaerospermum*, and *Penicillium chrysogenum* (Behera et al., 2017). Within *Bacteria*, several members of Firmicutes are able to degrade cellulose, including *Clostridium thermocellum*, *Ruminococcus flavefaciens*, and *Ruminococcus albus* (Flint et al., 2008). In addition, members of Actinobacteria, Bacteroidetes, Fibrobacteres, Pseudomonadaceae and Spirochaetae exhibit cellulolytic activity (Flint et al., 2008; Cardoso et al., 2012; Scully et al., 2013; Sravanthi et al., 2015). The enzyme systems for plant biomass breakdown of these microorganisms comprise various types of cellulolytic enzymes. Endoglucanases (EC 3.2.1.4) attack the cellulose chain randomly, exoglucanases or cellobiohydrolases (EC 3.2.1.91) attack at reducing or non-reducing ends of the cellulose chain, and beta-glucosidases (EC 3.2.1.21) hydrolyze the product cellobiose derived from the mentioned enzyme reactions (Nutt et al., 1998; Sadhu, 2013; Liu et al., 2018). Aerobic cellulolytic bacteria such as *Bacillus brevis* and *Pseudomonas fluorescens* secrete high amounts of extracellular cellulases (Singh and Kumar, 1998; Yamane and Suzuki, 1988), while anaerobic cellulolytic bacteria such as *C. thermocellum* and *R. albus* produce a complex and efficient cellulolytic machinery called cellulosome (Himmel et al., 2010; Behera et al., 2017). Cellulosomes consist of a scaffolding protein containing cohesin modules for incorporation of different enzymes, i.e., endoglucanase, carbohydrate-binding modules, and its complement module, dockerin. Cohesin-dockerin interaction is important for cellulosome assembly, as the cellulosome differs between bacterial species (Artzi et al., 2017). Although cellulolytic bacterial taxa from the gastrointestinal tract (hereinafter gut) of termites and herbivores such as cattle and panda have been intensively studied (Flint et al., 2008; Wilson, 2011; Nelson et al., 2013; Li et al., 2015), the gut bacterial community in the Eurasian beaver (*Castor fiber*) and the bacterial species responsible for the degradation of lignocellulose have not been reported.

The Eurasian beaver is a large semi-aquatic rodent that feeds on tree bark and some aquatic plants. It is the second largest rodent after the capybara (*Hydrochoerus hydrochaeris*) (Nolet and Rosell, 1998). The Eurasian beaver is one of two remaining species of the genus *Castor*, the other is the North American beaver, *Castor canadensis* (Rosell et al., 2005). The ability to digest hardwood is associated with gut-inhabiting microorganisms that facilitate the degradation of recalcitrant lignocellulosic material. Furthermore, the cellulolytic capability of the North American beaver gut system was studied (Hoover and Clarke, 1972; Wong et al., 2016, 2017; Armstrong et al., 2018). In addition, nitrogen-fixation by the Eurasian beaver gut microorganisms has been analyzed (Vecherskii et al., 2006, 2009). Recently, Gruninger et al. (2016) were able to classify bacterial and archaeal communities in the North American beaver gut, showing the dominance of Firmicutes, Bacteroidetes, and *Methanosphaera.* Similar information on the microbiome of the Eurasian beaver gut system was lacking prior to this study and is important to understand the ability of the beaver to digest hardwood.

The aim of this study was to characterize the bacterial community in the entire gastrointestinal tract of the Eurasian beaver (*C. fiber*). We used 16S rRNA gene amplicon analysis to reveal the bacterial community structure and diversity in the gut compartments (stomach, small intestine, cecum, and colon) of three beavers (female and male subadult, and male juvenile beaver). The analysis of each compartment of the beaver gut provided insights into changes of the bacterial community along the gut system. In this study, the bacterial communities of the gut system were directly recorded instead of using the fecal bacterial community as proxy for the gut bacterial community described in many other studies (Kohl et al., 2014; Ingala et al., 2018). We focused on cecum and colon as these compartments were mainly responsible for lignocellulolytic processes (Currier et al., 1960; Hoover and Clarke, 1972; Gruninger et al., 2016). In addition, the functional profiles of the bacterial communities in the different compartments were predicted. We also compared the Eurasian beaver gut bacterial communities to those of the North American beaver and several herbivorous animals.

## Materials and Methods

### Collection and Processing of Gut Content Samples

**FIGURE 1:**
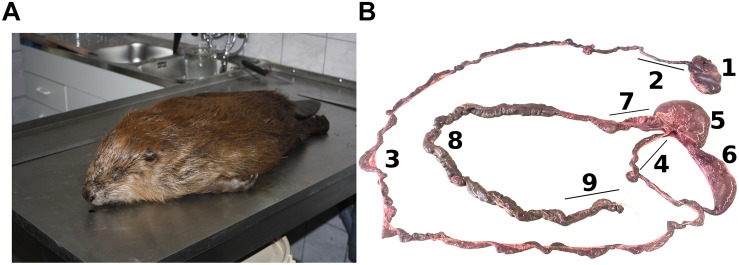
Beaver samples and digestive system of the beaver. **(A)** Carcass of the Eurasian beaver (courtesy of Antje Weber from *Büro Wildforschung* & *Artenschutz*, Jeggau 44a, 39649 Gardelegen, Germany). **(B)** The digestive system of the beaver showing the stomach (1), Duo (2), Jej (3), Ile (4), FC (5), BC (6), U.col (7), M.col (8), and L.col (9). One of rodent characteristic is the enlarged size of its cecum to ferment hardwood.

The Eurasian beaver is a protected animal in Germany. No animal was harmed or killed in the course of this study. Samples were taken from three beaver carcasses in Wittenberg, Saxony-Anhalt Germany, which died from traffic accidents in the “Biosphärenreservat Mittelelbe” (Figure 1A). The dead beavers were collected and stored at −20°C by personnel of the “Biosphärenreservat” before dissection. The beavers were juvenile (body weight 9.2 kg) and subadult male (body weight 17.5 kg), and subadult female (body weight 14.6 kg). The samples covered the entire gut system, from stomach to colon (Figure 1B). The pH values of all samples were recorded. Gut contents from the stomach (Sto), duodenum (Duo), jejunum (Jej), ileum (Ile), front cecum (FC), back cecum (BC), upper colon (U.col), middle colon (M.col), and lower colon (L.col) were extracted from the female subadult. The same gut contents were extracted from male subadult and juvenile beaver gut, except the duodenum part of male subadult and small intestine part (Duo, Jej, and Ile) of male juvenile beaver, as isolation of metagenomic DNA or PCR with the isolated DNA failed. Samples were stored at −80°C until further processing.

### DNA Extraction and Nucleic Acid Purification

Metagenomic DNA was extracted from approximately 100 mg wet gut content following the repeated bead-beating and column method (Yu and Morrison, 2004) with minor modifications. In brief, cells were lysed by bead-beating in 500 mM NaCl, 50 mM Tris–HCl, 50 mM ethylenediaminetetraacetic acid (EDTA) and 4% sodium dodecyl sulfate (SDS). The bead-beating process were done using a Micro Dismembrator (bbi-biotech GmbH, Berlin, Germany) for 30 s at 1,000 rpm. After bead-beating, most of the impurities and SDS were removed by precipitation with 10 M ammonium acetate. To remove protein and other contaminants the resulting nucleic acid pellet was further purified with Powerclean Pro kit following the instructions of the manufacturer (Qiagen GmbH, Hilden, Germany).

### Amplification of Bacterial 16S rRNA Genes

Amplification of bacterial 16S rRNA genes was performed by using primers targeting the V3 to V4 hypervariable region. The forward and reverse primer S-D-Bact-0341-b-S-17 (5′- CCTACGGGNGGCWGCAG-3′) and S-D-Bact-0785-a-A-21 (5′- GACTACHVGGGTATCTAATCC-3′) were utilized (Klindworth et al., 2013). Amplification was performed in a total volume of 50 μl containing 1 U Phusion high-fidelity DNA polymerase (Thermo Fisher Scientific, MA, United States), 10 μl of 5× Phusion GC Buffer, 0.2 mM of each primer, 10 mM dNTPs, 0.2 μl of 50 mM MgCl_2_, 5% DMSO and 25 ng of metagenomic DNA. Thermal cycling was carried out as follows: initial denaturation for 1 min at 98°C, followed by 25 cycles of 45 s at 98°C, 45 s at 60°C, 30 s at 72°C, and final elongation for 5 min at 72°C. The correct amplicon size (approximately 550 bp) was verified by agarose gel electrophoresis. Subsequently, the PCR products were purified using the magnetic bead kit NucleoMag 96 PCR as recommended by the manufacturer (Macherey-Nagel GmbH & Co. KG, Düren, Germany). Quantification of amplicons was conducted with the Qubit Fluorometer using the dsDNA HS assay kit (Invitrogen GmbH, Karlsruhe, Germany). Indices for Illumina sequencing were attached to the generated PCR products by using the Nextera XT index kit as recommended by the manufacturer (Illumina). Subsequently, the amplicons were sequenced by using the dual index paired-end approach for the MiSeq platform and v3 chemistry (2× 300 bp) as recommended by the manufacturer (Illumina).

### Bacterial Community Structure and Diversity Analysis

CASAVA data analysis software (Illumina) was used for demultiplexing and clipping of sequence adapters from raw sequences. Before removing sequences with an average quality score below 20 and unresolved bases with *split_libraries_fastq.py* from QIIME 1.9.1 (Caporaso et al., 2010), paired-end sequences were merged using PEAR v0.9.11 with default parameters (Zhang et al., 2014). Default settings of cutadapt 1.18 (Martin, 2011) were used for removal of non-clipped reverse and forward primer sequences. Generation of amplicon sequence variants (ASVs) (Callahan et al., 2017), chimera check, clustering, and creation of the abundance table were performed using VSEARCH v2.10.4 (Rognes et al., 2016). This included sorting by sequence length, size-filtering to ≥300 bp, and dereplication. Dereplicated ASVs were denoised using UNOISE3 with default settings, as well as chimera *de novo* removal with UCHIME. In addition, reference-based chimera removal was performed against the SILVA SSU v132 database (Quast et al., 2013). ASVs were clustered at 97% identity to generate operational taxonomic units (OTUs). Quality-filtered reads were mapped to OTUs to create OTU abundance tables. With *parallel_assign_taxonomy_blast.py* taxonomic classification of the OTU sequences against the SILVA database was done. *Filter_otu_table.py* was used for removal of chloroplasts, unclassified OTUs, and extrinsic domain OTUs. Finally, the lowest number of sequences by random subsampling (13,600 reads per sample) was used for sample comparison at the same surveying effort. Statistical test of alpha diversity (observed OTUs and phylogenetic diversity) from entire gut compartments and non-metric multidimensional scaling (NMDS) plots of the cecum and colon of the three beaver samples were calculated with the ampvis2 package in R (Andersen et al., 2018; R Core Team, 2018). The potential functional capabilities of gut bacterial communities were calculated with Tax4Fun package (Aßhauer et al., 2015) in R and SILVA database version 123.

### Comparison of Gut Bacterial Communities From Different Organisms

The 16S rRNA gene datasets used for comparison to that of the Eurasian beaver included bovine, giant and red panda, termite, North American beaver and human (Supplementary Table S1). Each 16S rRNA gene dataset was generated using different methods and approaches. In order to reduce bias when comparing these datasets, all datasets were preprocessed in a similar way to achieve comparable datasets and quality of 16S rRNA gene sequences. Datasets for which sequence quality scores were available (pandas, termites, Eurasian beaver, and North American beaver) were subjected to quality-filtering using *split_libraries.py* script from QIIME with default settings and minimal Q scores of 20. For bovine and human gut samples, according to the information of the authors, reads below 200 bp were excluded from subsequent analysis (Huttenhower et al., 2012; Jami et al., 2013). For the comparison with other 16S rRNA gene datasets obtained from cecum, rumen and fecal samples, we used only our beaver datasets from cecum and colon.

Open-reference OTU picking (*pick_open_reference_otus.py*) from QIIME was used to cluster the 16S rRNA genes of all studies. Open-reference OTU picking was performed with the non-redundant SILVA 132 SSU reference database at 97% sequence identity. The relative abundances at genus level calculated by QIIME *summarize_taxa.py* were used to perform multivariate analysis using Bray-Curtis dissimilarities. The community structure and NMDS plot were calculated with ampvis2 package in R Studio (Andersen et al., 2018). ANOSIM from vegan package in R Studio (Dixon, 2003) was performed to measure the similarity of bacterial communities across all samples.

### Accession Numbers

The 16S rRNA gene sequences were deposited in the National Center for Biotechnology Information (NCBI) Sequence Read Archive (SRA) under BioProject with the accession number PRJNA427255.

## Results and Discussion

### Diversity of Intestinal Bacterial Communities

**FIGURE 2:**
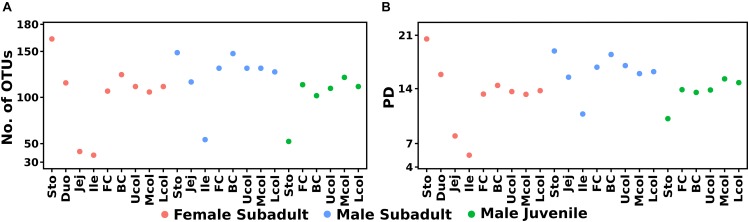
Diversity estimates of the bacterial community composition along the beaver gut samples, observed OTUs **(A)** and phylogenetic diversity **(B)**. The phylogenetic tree was midpoint-rooted using phangorn R package (Schliep, 2011) before alpha diversity calculation in ampvis2.

The Eurasian beaver gut bacterial communities in different parts of the gastrointestinal tract were characterized by 16S rRNA gene amplicon analysis. A total of 2,599,870 high-quality paired reads with an average read length of 450 bp were obtained. We identified a total of 277 unique OTUs across the entire dataset, which comprised 23 samples (Figure 2A). In general, the main fraction of the beaver gut bacterial community was covered by the surveying effort indicated by saturation of rarefaction curves (Supplementary Figure S1). The bacterial community of the subadult beaver stomach compartments was more diverse than that in cecum and colon (Figure 2B). This result is explained by considering that the stomach is the entry point of plant material and the associated diverse microbes into the digestive system. This is consistent with the results of Li et al. (2017), who showed that the stomach harbors a diverse bacterial community. A decrease of bacterial diversity was observed from stomach to the small intestine. The decrease of bacterial diversity might be related to the acidic pH values in the stomach, which serves as an entry filter for the gut bacterial community. The pH values in the stomach were 3.5, 5.1, and 5.7 for the female subadult, male subadult, and male juvenile beavers, respectively. In contrast to the subadult beaver, the stomach of the male juvenile beaver has the lowest diversity compared to its cecum and colon. Considering the age of the male juvenile, the bacterial community continues to develop in its gut system and stabilizes when the beaver reaches adulthood (Jami et al., 2013; Rodríguez et al., 2015). The diversity of the bacterial community in the cecum and colon of the three beavers varied. The communities in the cecum and colon of the male subadult beaver were more diverse than the communities in the subadult female and male juvenile beaver (Figure 2B).

**FIGURE 3:**
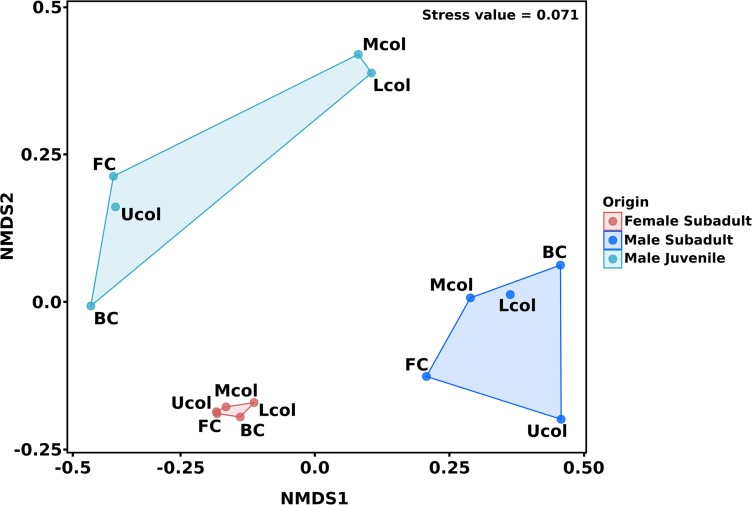
Non-metric multidimensional scaling (NMDS) of cecum and colon bacterial communities in the male and female Eurasian beaver. The ordination was calculated based on Bray-Curtis distance measure. The 15 gut compartment samples of cecum and colon were grouped according to beaver individuals before distance measure calculation.

The similarity in bacterial community composition of the cecum and colon was analyzed via NMDS (Figure 3). Differences in the bacterial communities were associated with the different compartments and individual beavers. The comparison of cecum and colon compartment based on the Bray-Curtis distance showed that the bacterial community of cecum and colon between the three beavers, as well as the bacterial community between cecum and colon of male beavers differed in both structure and abundance of OTUs. Similar results were obtained for the bacterial communities in cecum and colon of North American beavers (Gruninger et al., 2016).

### The Eurasian Beaver Gut Bacterial Community Is Dominated by Firmicutes and Actinobacteria

**FIGURE 4:**
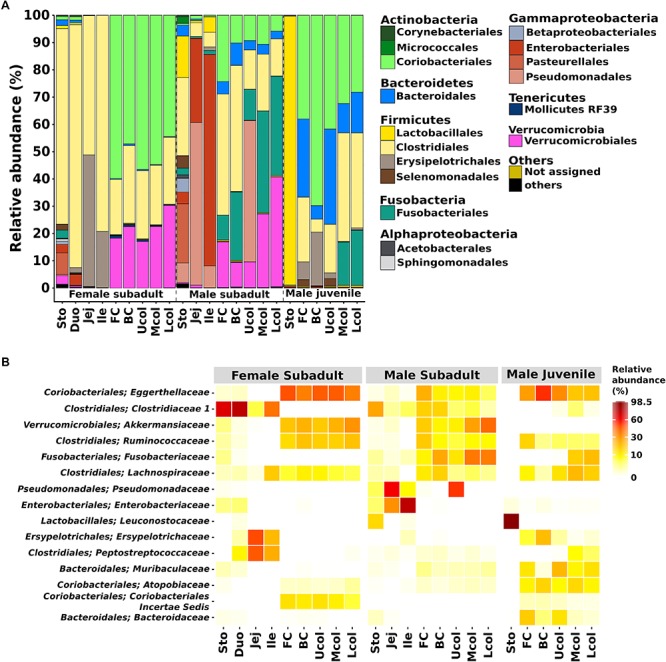
Bacterial community composition in the gut of Eurasian beaver. **(A)** Relative abundance of bacterial communities from male subadult, female subadult, and male juvenile beaver at order level. The figure represents the relative abundance of OTUs at 97% identity. **(B)** The top 15 most abundant bacterial from **(A)** were aggregated at family level (for details see Supplementary Table S2).

In the entire dataset, members of 8 bacterial phyla were detected. Most of the classified sequences belonged to Firmicutes (41.2%), Actinobacteria (23.6%), and Proteobacteria (12.6%). The other phyla were the Verrucomicrobia (9.5%), Fusobacteria (7.1%), Bacteroidetes (5.5%), and Tenericutes (0.1%) (Figure 4A). The dominance of Firmicutes and Actinobacteria in the Eurasian beaver gut system differed from the typical mammalian gut bacterial communities that primarily comprise Firmicutes and Bacteroidetes (Ley et al., 2008).

The Firmicutes and Actinobacteria were the dominant phyla in the gut system of the Eurasian beaver. The relative abundance of these phyla varied along the different gut compartments. The Firmicutes in the female subadult beaver were dominant in the stomach and small intestine (Duo, Jej, Ile) with 75.1% and more than 90% respectively, whereas in the cecum and colon it was less than 30%. We identified Firmicutes families known to comprise members with lignocellulolytic activity, including Clostridiaceae, Ruminococcaceae, and Lachnospiraceae (Figure 4B). The ability to degrade plant lignocellulose were reported for several species within these families such as *C. thermocellum*, *R. albus* and *Cellulosilyticum ruminicola* (Bayer et al., 1985; Lamed et al., 1987; Ding et al., 2001; Cai and Dong, 2010). Some of these, and other lignocellulolytic species from the same genera have been described as cellulosome formers (Lamed et al., 1987; Ding et al., 2001; Flint et al., 2008). The presence of *C. ruminicola* was recorded in the small intestine of the female subadult beaver. In contrast to the Firmicutes, the Actinobacteria were mainly present in the cecum and colon of all studied Eurasian beavers (Figure 4A). More than 60% of the detected Actinobacteria belonged to the Eggerthellaceae, with *Enterorhabdus* as the dominant genus (76.5–96.8%). The genus *Enterorhabdus* consists of three described species, *E. mucosicola*, *E. caecimuris*, and *E. muris*, which were isolated from mice intestine (Clavel et al., 2009, 2010; Lagkouvardos et al., 2016). To our knowledge, the presence of this genus has not been reported for gut systems outside mice and hamster (Clavel et al., 2014), suggesting that the members of this genus are host-specific. *Enterorhabdus* comprises aerotolerant bacteria that grow under anoxic conditions and utilize a variety of amino acid derivatives as energy source (Clavel et al., 2009). Although *Enterorhabdus* was present in high relative abundance in the gut system of the Eurasian beaver, this did not apply for the North American beaver as *Enterorhabdus* was not detected during our analysis. These results indicate that *Enterorhabdus* species are specifically associated with the Eurasian beaver.

The phylum Proteobacteria was detected in gut systems of both subadult beaver, especially in the male subadult beaver small intestine (jejunum and ileum, Figure 4A). Based on the 16S rRNA gene analysis of jejunum and ileum of the male subadult beaver, high relative abundances of *Pseudomonas* sp. (59.6 and 8%, respectively) and *Escherichia-Shigella* sp. (30.7 and 77.5%, respectively) were recorded. Members of the genus *Pseudomonas* are capable of using various organic and inorganic compounds,. Known cellulose degraders comprise *P. fluorescens var. cellulosa*, *P. nitroreducens*, and the newly isolated *P. coleopterorum* sp. *nov* (Yamane and Suzuki, 1988; Hazlewood et al., 1992; Huang et al., 2012; Menéndez et al., 2015). Some of the *Pseudomonas* species also play a role in nitrogen fixation and metabolism such as *P. aeruginosa* (Wu et al., 2005), and *P. stutzeri* (Xie et al., 2006). Thus, *Pseudomonas* could also play an important role in the nitrogen metabolism of the gut system. In addition, members of *Escherichia and Shigella*, which are closely related and share many common characteristic (Devanga Ragupathi et al., 2018), were present. They are known to contaminate water bodies (Jun et al., 2016; Probert et al., 2017). The presence of *Escherichia-Shigella* in the beaver gut might originated from a contaminated water source in the habitat of the beaver.

The phylum Verrucomicrobia was detected in the cecum and colon of both subadult beavers (9–17.1% and 30.2–40.3%, respectively), but was not detected in male juvenile beaver samples. Based on the study of *Akkermansia* sp., the colonization of Verrucomicrobia will increase and reach its maximum abundance when the host becomes adult (Derrien et al., 2008). Members of Verrucomicrobia were also reported to inhabit the human gut (Flint et al., 2012), the bovine rumen (Li et al., 2012), and the North American beaver gut (Gruninger et al., 2016). The Verrucomicrobia in our beavers consisted solely of *Akkermansia*. To date, *Akkermansia* comprises two described species, *A. muciniphila* and *A. glycaniphila* isolated from a human fecal sample (Derrien et al., 2004), and a python fecal sample (Ouwerkerk et al., 2016), respectively. *A. muciniphila* plays an essential role in the human gut and supports maintaining a healthy metabolic status (Dao et al., 2016).

The phylum Fusobacteria, which was mainly represented by *Fusobacterium*, is abundant in both cecum (9–25%) and colon (11.3–37.2%) of the male subadult beaver, and the colon (15.8– 20.1%) of male juvenile beaver (Figure 4A). The presence of *Fusobacterium* in the gut system is often associated with pathogenicity, i.e., *F. necrophorum*, causes Lemierre’s disease (Riordan, 2007) and *F. nucleatum* is enriched in patients with chronic gut inflammation (Allen-Vercoe et al., 2011). In contrast to the previous two species, *F. varium* provides butyrate and acetate, which are important to maintain a healthy colon (Potrykus et al., 2007). In the cecum and colon of male juvenile beaver, the phylum Bacteroidetes was present in high relative abundance (5–34.9%), and mainly represented by Muribaculaceae (part of Bacteroidales S24-7) and Bacteroidaceae. Previous studies in 57 unique animal species related to Bacteroidales S24-7 showed that 96% of the animals harboring Bacteroidales S24-7 were herbivores or omnivores mainly consuming herbivorous food (Ormerod et al., 2016). Similar to Bacteroidales S24-7, members of Bacteroidaceae were enriched in the gut systems of Korean adolescents, who consumed mainly plant-based and fermented foods (Jang et al., 2017). Members of the dominant genus *Bacteroides* are among the most common taxa of the human gut bacterial community (Ramakrishna, 2013). Members of this genus were reported to exhibit cellulolytic activity, i.e., *B. cellulosilyticus* in human gut (Robert et al., 2007) and *B. luti* in methanogenic sludge (Hatamoto et al., 2014). Further analysis of the bacterial community in the Eurasian beaver gut is required to analyze the shift in Bacteroidetes abundance from juvenile to subadult beavers.

Based on bacterial community composition, it is indicated that the degradation of lignocellulosic plant material occurred within the cecum and colon compartment, as putative cellulolytic members of Ruminococcaceae, and Lachnospiraceae are present almost exclusively in cecum and colon. This also suggests that these have colonized the beaver cecum and colon from an early age on in order to prepare juvenile beavers to adapt to lignocellulosic food after weaning.

### Potential Functional Capabilities of the Beaver Gut Microbiome

We used Tax4Fun to predict functional profiles from our 16S rRNA gene datasets (Aßhauer et al., 2015). The Tax4Fun prediction has been shown to provide a good correlation of functional profiles with metagenomic profiles derived from direct sequencing (Aßhauer et al., 2015). In addition, Tax4Fun prediction returned a high coverage of mammalian gut bacterial community, a functional profile could be predicted for about 95% of the OTUs (Aßhauer et al., 2015). So far, functional profiles using Tax4Fun have been predicted from 16S rRNA gene datasets derived from various environments (Kaiser et al., 2016; Hasegawa et al., 2017; Wemheuer et al., 2017; Berkelmann et al., 2018; Xu et al., 2018).

**FIGURE 5:**
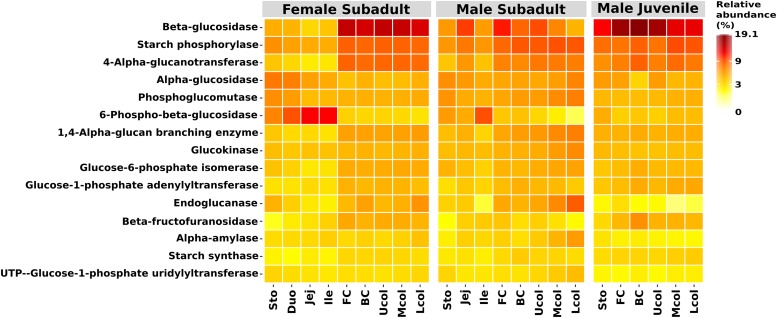
Functional prediction of the top 15 functions in the KEGG categories starch and sucrose metabolism. Shown are the relative abundances. Functional prediction was performed with Tax4Fun (Aßhauer et al., 2015).

We were able to assign functional profiles for 74.85% of our OTUs. Based on the ability of the beaver microbiome to degrade hardwood, we focused our analysis on metabolic functions associated with cellulolytic activity derived from the KEGG pathway database. We recorded increased abundance of endoglucanase and beta-glucosidase genes in the cecum and colon of the three beavers (Figure 5). Since plant material also consists of storage polysaccharides, i.e., starch, the predicted genes associated with starch modification (starch phosphorylase and 4-alpha-glucanotransferase) were also abundant in the cecum and colon (Figure 5). All genes encoding the above-mentioned enzymes were also present in the stomach and small intestine but in lower relative abundance than in the cecum and colon. This could be the result of coprophagy in the beavers, since beavers require nutrients from microbial metabolism. The presence of endoglucanase, beta-glucosidase, starch phosphorylase, and 4-alpha-glucanotransferase together with the abundance of cellulolytic bacteria from the phylum Firmicutes in the cecum and colon compartment indicate that the breakdown of lignocellulosic plant material takes place in these gut compartments.

### Eurasian Beaver Gut Microbiome in Comparison With North American Beaver and Other Herbivores

We conducted comparative analyses of the gut bacterial communities between Eurasian (Eu) beaver and two North American (NA) beavers. which derived from different studies, NA_*Grun*_ and NA_*Wong*_ (Gruninger et al., 2016; Wong et al., 2016, respectively). Additionally, the gut bacterial communities of other herbivorous animals, such as bovine (Jami et al., 2013), giant and red panda (Li et al., 2015), termite (Dietrich et al., 2014), and human (Huttenhower et al., 2012) were included (Supplementary Table S1). To create comparable bacterial community 16S rRNA gene datasets of the different animals, the datasets were divided into two groups: group A comprised the datasets from Eu beaver cecum, NA_*Grun*_ beaver cecum, bovine rumen, and termite hindgut as these compartments harbor lignocellulolytic bacterial communities; and group B comprised the datasets from fecal samples of giant and red pandas, NA_*Wong*_ beaver, and human, in addition to the colon of Eu and NA_*Grun*_ beaver, which is comparable to fecal samples. The different grouping of rumen/cecum and fecal samples was intended, as the bacterial community derived from fecal material cannot be directly regarded as representative for the gut bacterial community (Kohl et al., 2014; Ingala et al., 2018).

**FIGURE 6:**
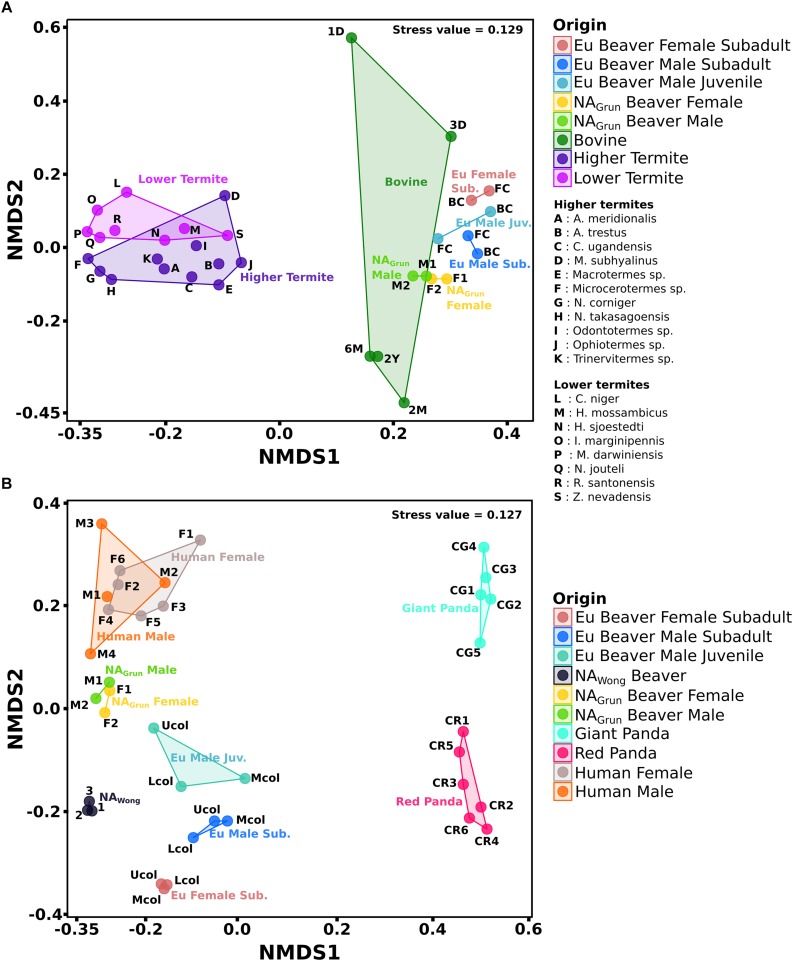
NMDS of gut bacterial communities of herbivorous animals and humans. The combined datasets separated based on sample source. **(A)** Group A consists of cecum, rumen, and termite gut samples. **(B)** Group B consists of colon and fecal samples. The addition of human data was to compare with non-herbivore gut bacterial community. The distance between the ordination of one sample to another indicates the dissimilarity of the gut bacterial community between those samples (for details see Supplementary Table S3).

The comparison of group A (Figure 6A) shows that the cecum bacterial community of the Eu beavers differed from that of the North American beavers (NA_*Grun*_) (*R* = 0.881, *P* < 0.05), the bovine (*R* = 0.908, *P* < 0.05), the higher termites (*R* = 0.976, *P* < 0.05), and the lower termites (*R* = 0.998, *P* < 0.05). This is mainly due to differences in the composition of the dominant phyla. The NA_*Grun*_ cecum and bovine rumen, and partly also the gut of higher termites such as *A. trestus*, *Macrotermes* sp., and *Odontotermes* sp., were dominated by Firmicutes and Bacteroidetes as previously reported (Flint et al., 2012; Supplementary Figure S2). This was distinct from the Eu beaver cecum in which members of the Actinobacteria were more abundant than members of Bacteroidetes. The Firmicutes families, Ruminococcaceae and Lachnospiraceae, were present in both Eu and NA beaver cecum as well as in bovine rumen, and the majority of termite gut systems (Supplementary Figure S3), suggesting that it is common family associated with herbivorous gut microbiomes (Jami and Mizrahi, 2012; Ferrario et al., 2017; Singh et al., 2017).

The Streptococcaceae was the major family of the rumen bacterial community of 1-day old bovine (Supplementary Figure S3). In accordance with previous studies on mammals this family is among the earliest colonizers of the infant gut, transmitted from the mother by breastfeeding (Rodríguez et al., 2015). For bovine aged 2 months to 2 years, members of Bacteroidetes, the Prevotellaceae, dominate in the rumen (59%) and play an important role in the intake of carbohydrates (Kim et al., 2011; Ramakrishna, 2013). The presence of Prevotellaceae has also been detected in the cecum of Eu and NA beaver, but in relative abundances of less than 10%. The abundance of the dominant Actinobacteria family, Eggerthellaceae, was observed only within the Eu beaver cecum (Supplementary Figure S2), which supports our earlier hypothesis that the member of this family *Enterorhabdus* is specific for the Eu beaver gut. The phylum Spirochaetae was also abundant in several termite gut systems. Spirochaetae account for up to 50% of all prokaryotes present in some termites, and are involved in the degradation of cellulose and hemicellulose (Lilburn et al., 1999; Droge et al., 2006; Dubinina et al., 2015; Sravanthi et al., 2015).

The feces-derived bacterial communities (group B, Figure 6B) showed that the bacterial community of the Eu beaver differed from that of the giant panda (*R* = 1, *P* < 0.05), red panda (*R* = 0.986, *P* < 0.05), NA_*Grun*_ beaver (*R* = 0.889, *P* < 0.05), NA_*Wong*_ beaver (*R* = 0.801, *P* < 0.05), and human (*R* = 0.974, *P* < 0.05). The Firmicutes were generally discovered in high abundance in all samples, particularly in the giant and red pandas, in which Firmicutes account for more than 80% of the total bacterial community (Supplementary Figure S4). The exceptions were NA_*Wong*_ (less than 10%) and human male 3 (2.1%). Between the two colon samples of Eu and NA_*Grun*_ beavers, Ruminococcaceae and Lachnospiraceae were the common Firmicutes families (Supplementary Figure S5). These families are also generally present in human fecal samples. As already mentioned, Ruminococcaceae and Lachnospiraceae facilitate breakdown of plant cell walls in the host gut and are common in gut systems of herbivores. Since humans are omnivores, a dietary change to a fiber-rich diet increases the abundance of Ruminococcaceae and Lachnospiraceae (Muegge et al., 2011; Singh et al., 2017). The giant and red panda fecal samples were dominated by the families Streptococcaceae and Clostridiaceae 1, respectively. Members of the Streptococcaceae were not present in the Eu beaver colon samples, but members of Clostridiaceae 1 were present below 7% relative abundance. The giant pandas are known to possess digestive tracts like carnivores, with a simple stomach, degenerated cecum, rapid transit time, and gut microbiome similar to those of bears (Li et al., 2015; Xue et al., 2015). On the other hand, the bacterial community of the red panda gut did not differ in diversity compared to the giant panda (Supplementary Figure S5), although the bacterial diversity in the gut of the wild red panda was higher than that of the captive red panda (Kong et al., 2014). Members of the Verrucomicrobia were discovered in human and all beaver fecal samples (Supplementary Figure S4). The low abundance of Verrucomicrobia in both North American beaver and human gut might be related to a poor health status of the host, as the abundance of *Akkermansia* sp. has been inversely correlated to several disease states (Geerlings et al., 2018). Members of the Fusobacteria were abundant in Eu and NA_*Wong*_ beaver, and also in human male 2 (Supplementary Figure S5). Since the Fusobacteria have been reported to be abundant in the gut of marine carnivorous mammals, their presence in the Eu beaver gut needs to be further investigated in order to ascertain their role in the gut system. Interestingly, the fecal sample of NA_*Wong*_ beaver and the upper colon of the male subadult Eu beaver showed a high abundance of Pseudomonadaceae (Supplementary Figure S5). Although some species of *Pseudomonas* are able to degrade cellulose (Lejeune et al., 1986; Hazlewood et al., 1992; Huang et al., 2012), their presence is not common in other gut systems of other beaver.

## Conclusion

In this study, we have uncovered the bacterial community structure and diversity within the Eurasian beaver gut system. The gut bacterial community was dominated by Firmicutes and Actinobacteria. The presence and abundance of some phyla could be associated with sex, i.e., Fusobacteria were detected in both male beavers but not in the female. An association with age of members of the Verrucomicrobia was indicated, which were higher abundant in subadult beavers than in the juvenile beaver. However, further studies of Eurasian beaver gut microbial communities are necessary to confirm these trends. Several taxa involved in degradation of lignocellulose have been identified. The presence of Lachnospiraceae and Ruminococcaceae in the cecum and colon of the beaver indicated that plant cell wall breakdown is mainly performed in these compartments, which is also indicated for the North American beaver. The predicted functional profiles showed an increased relative abundance of genes necessary for cellulose breakdown in cecum and colon, which is in accordance with the presence of potential cellulolytic bacterial species. In comparison to its North American relative, the Eurasian beaver has a higher relative abundance of Actinobacteria and Verrucomicrobia, and lower abundance of Bacteroidetes in the gut system. The abundance of unclassified Clostridiaceae 1, Lachnospiraceae, and Ruminococcaceae in the cecum and colon of the beaver as well as unclassified Bacteroidaceae in juvenile beaver suggest the presence of novel species exhibiting cellulolytic activity. Further studies on the expressed cellulolytic genes and enzyme activities of these communities are needed to unravel the lignocellulose breakdown by the Eurasian beaver gut microbiome.

## Data Availability

The datasets generated for this study can be found in the National Center for Biotechnology Information (NCBI) Sequence Read Archive (SRA) under the project accession PRJNA427255.

## Ethics Statement

The Eurasian beaver is a protected animal in Germany. No animal was harmed or killed in the course of this study. Gut samples were taken from three beavers, which were found dead in the Biosphärenreservat Mittelelbe by personnel of the Biosphärenreservat after being hit by cars. The beavers died by accidents.

## Author Contributions

RD designed and conceived the study. RP and TB carried out the experimental work. RP and DS prepared and analyzed the data. RP, DS, and RD wrote the manuscript. All authors interpreted the results and approved the final version of the manuscript.

## Conflict of Interest Statement

The authors declare that the research was conducted in the absence of any commercial or financial relationships that could be construed as a potential conflict of interest.

## Supplementary Material

FIGURE S1 Rarefaction curves of gut compartments from all beaver samples. The 16S rRNA gene sequences were randomly subsampled to 13,600 reads.

FIGURE S2 The bacterial community of group A based on cecum/rumen and gut samples of termites at phylum level. The top 10 phyla are shown. The color corresponds to the relative abundance. FS, female subadult beaver; MS, male subadult beaver; MJ, male juvenile beaver; FC, front cecum; BC, back cecum. Bovine 1D, 1 day old; 3D, 3 days old; 2M, 2 months old; 6M, 6 months old; 2Y, 2 years old.

FIGURE S3 The bacterial community of group A based on cecum/rumen and gut samples of termites at the family level. The top 15 families are shown. The color corresponds to the relative abundance. FS, female subadult beaver; MS, male subadult beaver; MJ, male juvenile beaver; FC, front cecum; BC, back cecum. Bovine 1D, 1 day old; 3D, 3 days old; 2M, 2 months old; 6M, 6 months old; 2Y, 2 years old.

FIGURE S4 The bacterial community of group B based on colon and fecal sample at the phylum level. The top 10 phyla are shown. The color corresponds to the relative abundance. FS, female subadult beaver; MS, male subadult beaver; MJ, male juvenile beaver; Ucol, upper colon; Mcol, middle colon; Lcol, Lower colon; CG, captive giant panda; CR, captive red panda.

FIGURE S5 The bacterial community of group B based on colon and fecal sample at the family level. The top 15 families are shown. The color corresponds to the relative abundance. FS, female subadult beaver; MS, male subadult beaver; MJ, male juvenile beaver; Ucol, upper colon; Mcol, middle colon; Lcol, Lower colon; CG, captive giant panda; CR, captive red panda.

TABLE S1 Dataset of 16S rRNA gene amplicon libraries used in this study.

TABLE S2 OTU table at 97% sequence identity at species level. Samples were randomly subsampled to 13,600 reads and absolute counts are shown.

TABLE S3 OTU table at 97% sequence identity at species level. Absolute counts are shown.
