# Supplementary figures and images for: First Insights Into Bacterial Gastrointestinal Tract Communities of the Eurasian Beaver (Castor fiber)
Source: Front Microbiol. 2019 Jul 25;10:1646. doi: 10.3389/fmicb.2019.01646 (PMC6690062; doi:10.3389/fmicb.2019.01646)

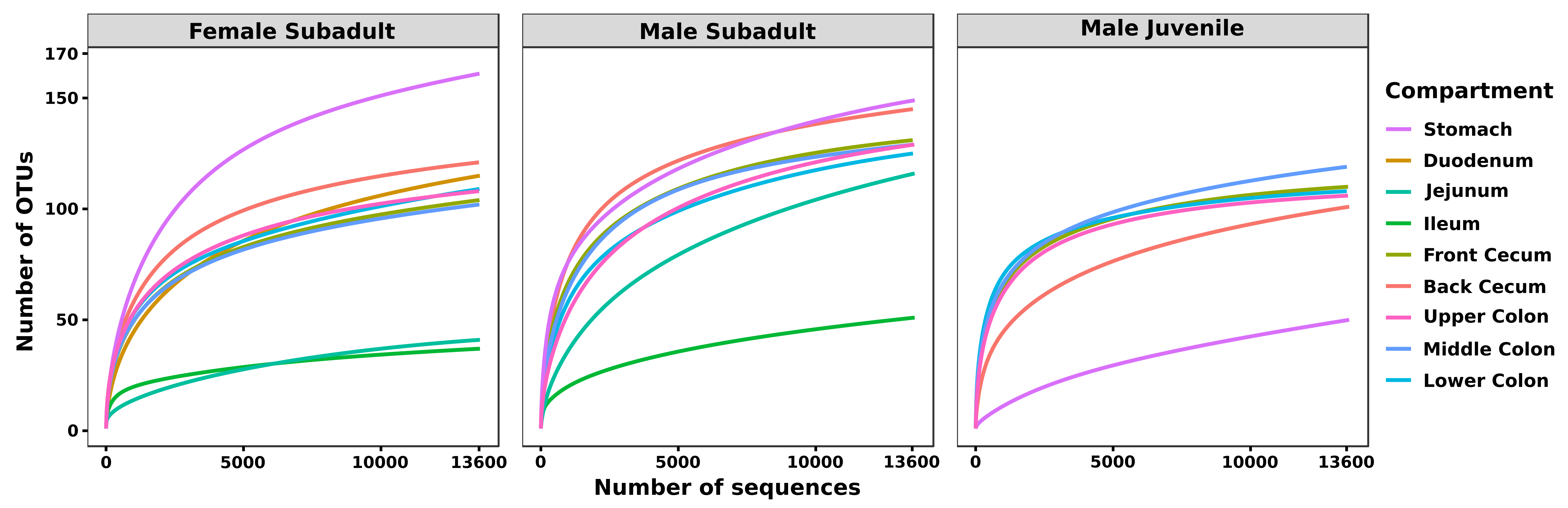

Supplement: FIGURE S1 — Rarefaction curves of gut compartments from all beaver samples. The 16S rRNA gene sequences were randomly subsampled to 13,600 reads. [file Image_1.TIF]

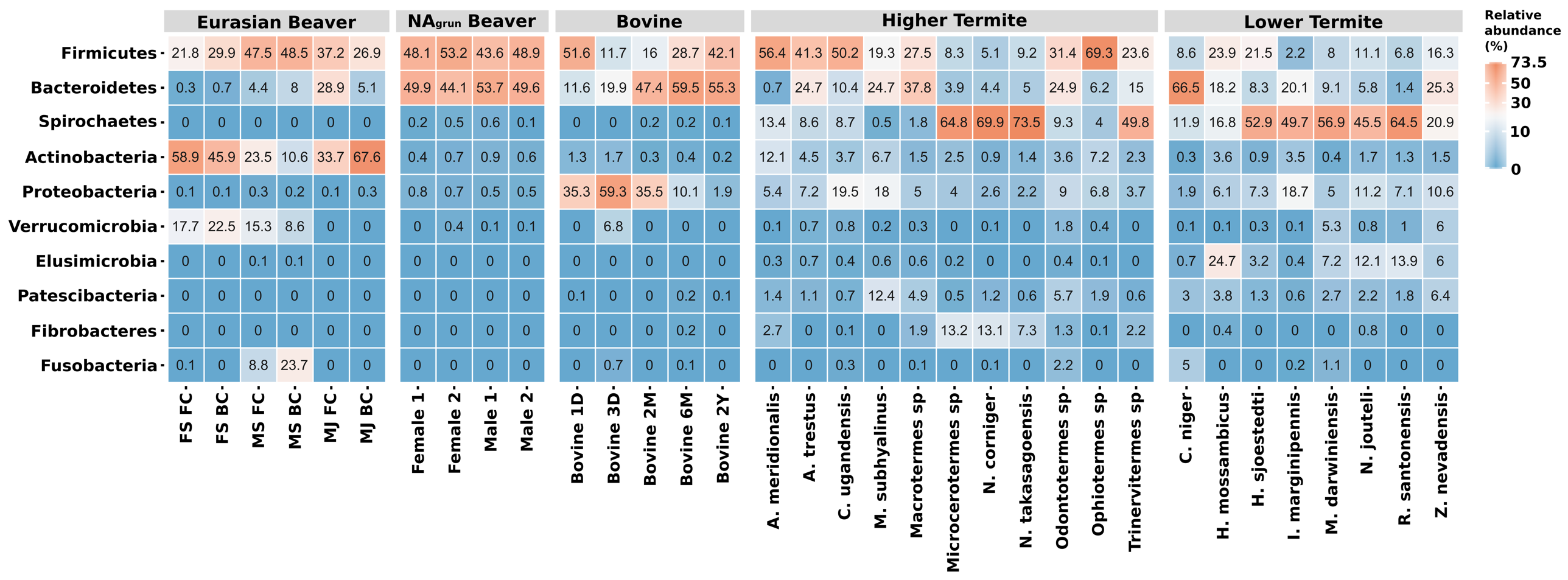

Supplement: FIGURE S2 — The bacterial community of group A based on cecum/rumen and gut samples of termites at phylum level. The top 10 phyla are shown. The color corresponds to the relative abundance. FS, female subadult beaver; MS, male subadult beaver; MJ, male juvenile beaver; FC, front cecum; BC, back cecum. Bovine 1D, 1 day old; 3D, 3 days old; 2M, 2 months old; 6M, 6 months old; 2Y, 2 years old. [file Image_2.TIF]

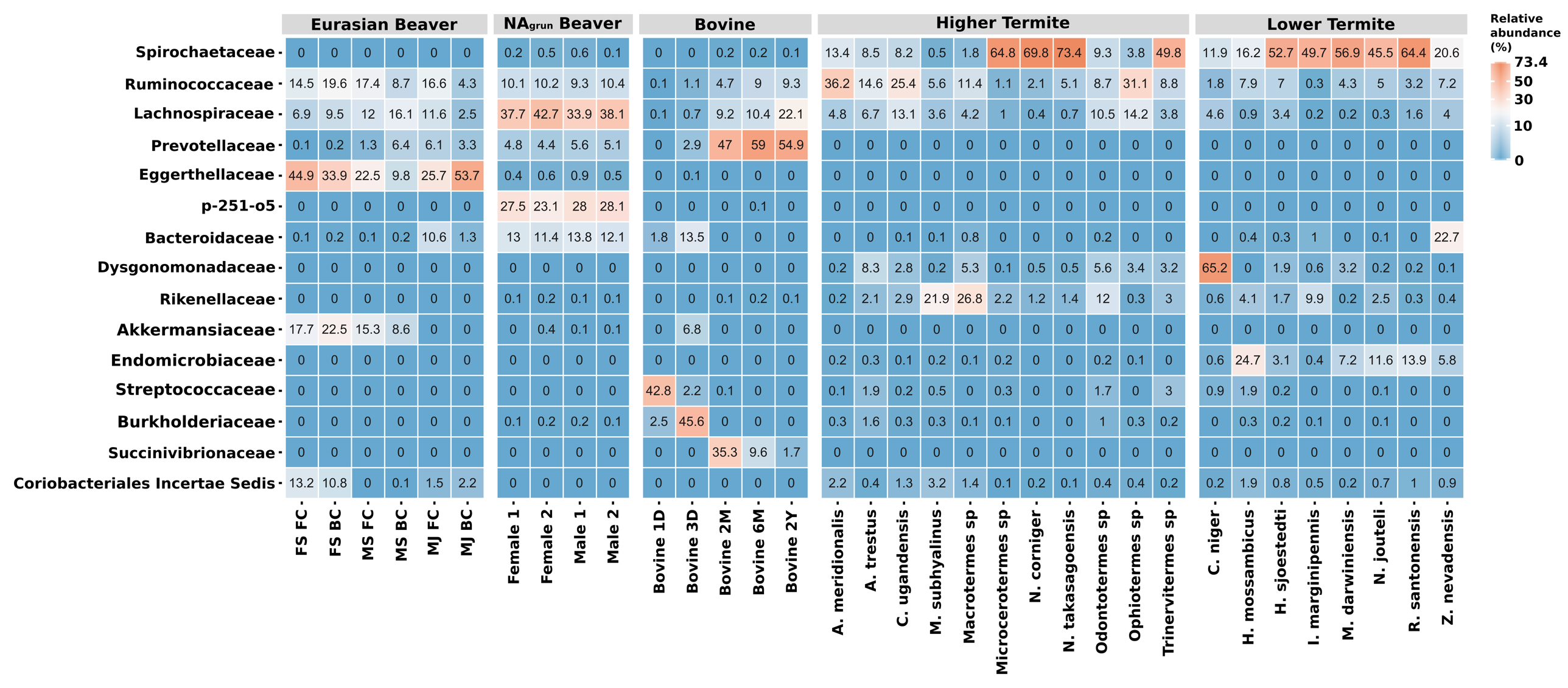

Supplement: FIGURE S3 — The bacterial community of group A based on cecum/rumen and gut samples of termites at the family level. The top 15 families are shown. The color corresponds to the relative abundance. FS, female subadult beaver; MS, male subadult beaver; MJ, male juvenile beaver; FC, front cecum; BC, back cecum. Bovine 1D, 1 day old; 3D, 3 days old; 2M, 2 months old; 6M, 6 months old; 2Y, 2 years old. [file Image_3.TIF]

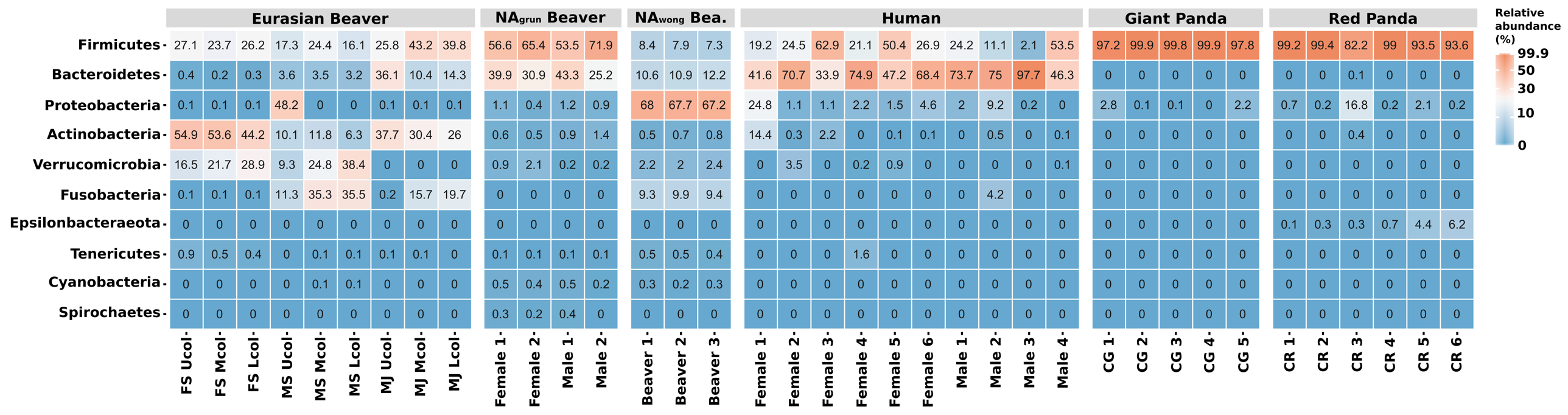

Supplement: FIGURE S4 — The bacterial community of group B based on colon and fecal sample at the phylum level. The top 10 phyla are shown. The color corresponds to the relative abundance. FS, female subadult beaver; MS, male subadult beaver; MJ, male juvenile beaver; Ucol, upper colon; Mcol, middle colon; Lcol, Lower colon; CG, captive giant panda; CR, captive red panda. [file Image_4.TIF]

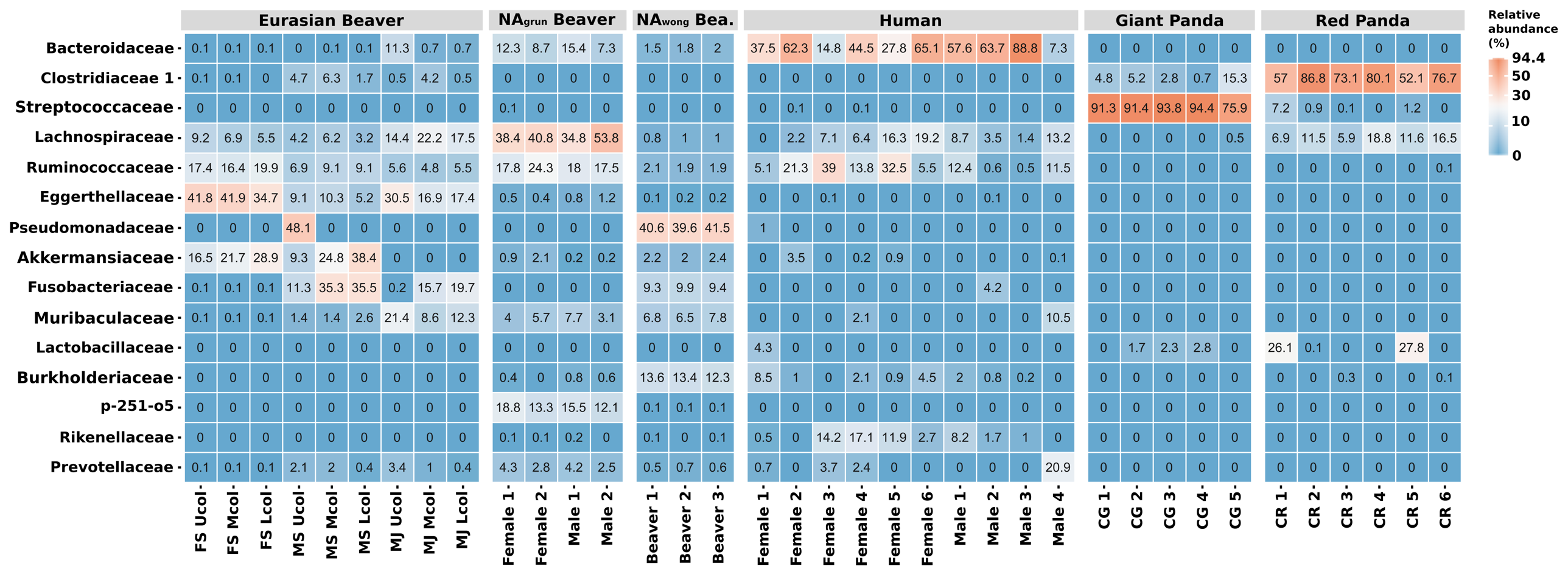

Supplement: FIGURE S5 — The bacterial community of group B based on colon and fecal sample at the family level. The top 15 families are shown. The color corresponds to the relative abundance. FS, female subadult beaver; MS, male subadult beaver; MJ, male juvenile beaver; Ucol, upper colon; Mcol, middle colon; Lcol, Lower colon; CG, captive giant panda; CR, captive red panda. [file Image_5.TIF]
