# Supplementary material for: First Insights Into Bacterial Gastrointestinal Tract Communities of the Eurasian Beaver (Castor fiber)
Source: Front Microbiol. 2019 Jul 25;10:1646. doi: 10.3389/fmicb.2019.01646 (PMC6690062; doi:10.3389/fmicb.2019.01646)
Supplement: TABLE S1 — Dataset of 16S rRNA gene amplicon libraries used in this study. [file Table_1.DOCX]

**Table S1.** Dataset of 16S rRNA gene amplicon libraries used in this study**.**

| **Study** | **No. of samples** | **Sample type** | **16S rRNA gene region** | **Sequencing platform** | **Accession numbers^g^ and** r**eferences** |
| --- | --- | --- | --- | --- | --- |
| Bovine | 21^a^ | Rumen content | V2 – V3 | Roche 454 | 4514864.3 - 868.3 _(MG-RAST)_ (Jami et al., 2013) |
| Eurasian beaver | 23^b^ | Gut content | V3 – V4 | Illumina MiSeq | PRJNA427255 (this study) |
| Human | 10^c^ | Feces | V3 – V5 | Roche 454 | SRS016152, 016437, 021664, 023914, 042290, 042703, 052196, 055137, 064276, 065665 (Huttenhower et al., 2012) |
| North American beaver _(Grun)_ | 4^d^ | Gut content | V1 – V3 | Illumina MiSeq | SRP069012, 069014 (Gruninger et al., 2016) |
| North American beaver _(Wong)_ | 3 | Feces | V5 – V8 | Roche 454 | SRR2905007 (Wong et al., 2016) |
| Panda | 11^e^ | Feces | V1 – V3 | Roche 454 | SRR1766294 (Li et al., 2015) |
| Termite | 19^f^ | Gut content | V3 – V4 | Roche 454 | SAMN02228083 – 101 (Dietrich et al., 2014) |

^a^ Samples grouped to 5 age group: 1 day (n=3), 3 days (n=3), 2 months (n=5), 6 months (n=5), 2 years (n=5);

^b^ gut compartment samples grouped to 3 individuals: male juvenile (n=6), male subadult (n=8), female subadult (n=9);

^c^ samples grouped to 2 sex group: male (n=4), female (n=6);

^d^ samples grouped to 2 sex group: male (n=2), female (n=2);

e samples grouped to 2 panda type: giant panda (n=5), red panda (n=6);

^f^ samples grouped to 2 class: lower termite (n=8), higher termite (n=11);

^g^ unless noted, all sample sequences were obtained from the NCBI server.
